# Expert Panel Guidelines for Hybrid CaHA‐CMC/CPM‐HA Fillers in the Mexican Population

**DOI:** 10.1111/jocd.70548

**Published:** 2025-11-17

**Authors:** Victoria de la Fuente García, Susana Sil Zavaleta, María Guadalupe Lozada Arenas, Javier Ruiz Ávila, Socorro Isela Méndez Baca, Jorge Torres García, Alejandro García Balbas, Hiram Rogelio Elizondo Vilchez, Javier Reynoso Vázquez, Randall Alfonso Herrera Lozano, Nabil Fakih‐Gomez

**Affiliations:** ^1^ Levels High‐End Dermatology Mexico City Mexico; ^2^ Hands and Skin, Hospital Angeles Mexico City Mexico; ^3^ Clinique D' Ozono Mexico City Mexico; ^4^ Dermedica Clinic Mexico City Mexico; ^5^ Isela Mendez Dermatology Clinic Mexico City Mexico; ^6^ Allure Antiaging Clinic Saltillo Mexico; ^7^ Dr. AGB Clinic Queretaro Mexico; ^8^ Dr. Hiram Elizondo Clinic Mexico City Mexico; ^9^ The Health Center Puebla Mexico; ^10^ RH Clinic Leon Guanajuato Mexico; ^11^ Department of Facial Plastic and Cranio‐Maxillo‐Facial Surgery Fakih Hospital Khaizaran Lebanon

**Keywords:** CaHA, CaHA‐CMC, calcium hydroxylapatite, CPM, CPM‐HA, hyaluronic acid, hybrid filler, Mexicans, recommendations

## Abstract

**Introduction:**

Combination of calcium hydroxylapatite‐carboxymethylcellulose (CaHA‐CMC) and hyaluronic acid (HA) in a single syringe has shown effectiveness and safety. In light of the growing popularity of hybrid fillers in aesthetic medicine, there is a need to establish treatment guidelines, especially focusing on diverse populations, including Mexicans.

**Methods:**

By combining the clinical expertise of 10 Mexican injectors with scientific evidence, recommendations were established for the use of hybrids in treating patients from a Mexican population. The panel reached consensus on 10 topics: skin preparation, anesthetic use, hybrid filler preparation, syringe choice, injection protocol by anatomical zone, duration of treatment, complications/adverse events, patient perspective, treatment costs and future perspectives.

**Results:**

Regardless of the CPM‐HA or anatomic area, a 1:1 ratio of CaHA‐CMC to CPM‐HA, was the preferred ratio for filler preparation and was administered subcutaneously using fanning and retro‐injection techniques. Almost all experts (9/10, 90%) believed the clinical outcomes obtained with hybrid fillers and the reduced treatment time (number of visits required) justify the increased cost. Main factors in driving increased adoption of hybrid treatments in clinical practice were high patient satisfaction, clinical efficacy, innovation, and prolonged duration of results. The choice of CPM‐HA for the hybrid filler should be guided by skin quality, degree of sagging, volume loss, underlying anatomy, and patient expectations.

**Conclusions:**

This paper provided recommendations regarding the preparation and injection of CaHA‐CMC and CPM‐HA combinations across different anatomical regions. These contributions aim to support clinical decision‐making, optimize outcomes, and improve patient safety in aesthetic treatments.

## 1 Introduction

The hybrid filler technique in aesthetic and regenerative medicine involves the combination of different injectable products; typically calcium hydroxylapatite‐carboxymethylcellulose (CaHA‐CMC; Radiesse, Merz North America Inc., Franksville, WI, USA) has been mixed with one of the cohesive polydensified matrix hyaluronic acid (CPM‐HA) fillers (Belotero collection; Anteis S.A., Plan‐les‐Ouates, Switzerland, a company of the Merz Aesthetics group) to be administered simultaneously in a single syringe. In addition to offering an innovative approach for addressing various age‐related changes, such as volume loss, alterations in the extracellular matrix (ECM), and decreased elasticity, hybrid fillers may also enhance the patient experience by reducing the need for multiple injections. Moreover, if the blended products have complementary mechanisms of action, the resulting hybrid filler may address multiple pathophysiologic aspects of aging, thereby expanding the scope of treatment. For instance, in the CaHA:CPM‐HA blend, the CPM‐HA gel provides an immediate volumizing effect, compensating for the early volume loss secondary to the resorption of the carboxymethylcellulose (CMC) gel carrier [1], whereas the biostimulatory and ECM regenerating properties of CaHA‐CMC extend the duration of the filler treatment [2]. Furthermore, combining the strong structural support of CaHA‐CMC with the smooth texture of CPM‐HA creates a gel that is more pliable, moldable, and easier to administer than undiluted CaHA‐CMC alone [3]. This combination has shown effectiveness and safety in the treatment of various facial areas [1, 3, 4, 5, 6, 7, 8]. No histologic and immunohistologic signs of inflammation or disturbance of tissue trophism following simultaneous CaHA‐CMC and CPM‐V injection have been observed [1, 6]. Nonetheless, ongoing research is required to continue monitoring and reporting on filler safety.

Although hybrid fillers are gaining popularity in aesthetic medicine [9], there remains a growing need to establish treatment guidelines and protocols, driven by the limited and still‐developing scientific evidence. Defining optimal practices, appropriate product volumes, and safe injection techniques is essential to improve safety and achieve consistent aesthetic outcomes. Product selection, blending ratio and injection depth require carefully tailoring to the treatment area to achieve specific desired effects. At present, only one study has proposed standardized protocols for hybrid filler application in aesthetic medicine [4]; however, these recommendations were based on treatments to a Middle Eastern and European population (based in Lebanon and the Netherlands) and its applicability to the Mexican population may be limited given the numerous anatomical and clinical differences between individuals of these diverse populations. To address the need for region‐specific recommendations, we conducted an expert panel discussion to devise recommendations for hybrid fillers treatments (specifically CaHA‐CMC combined with CPM‐HA, for face, neck, décolletage, and hand rejuvenation) in the Mexican population.

## 2 Methods

A group of 10 Mexican physicians specialized in dermatology, plastic surgery, or aesthetic medicine, each with a minimum of 3 years of experience using hybrid products and recognized in the field of aesthetic medicine, was identified and invited to virtually discuss the hybrid filler technique combining CaHA‐CMC and CPM‐HA.

The discussion was focused on four hybrids, created by combining CaHA‐CMC with 4 different CPM‐HA gels: CPM‐R (Belotero Revive containing 20 mg/mL of HA; Anteis S.A., Plan‐les‐Ouates, Switzerland, a company of the Merz Aesthetics group), CPM‐B (Belotero Balance, 22.5 mg/mL HA, Anteis S.A.), CPM‐I (Belotero Intense, 25.5 mg/mL HA; Anteis S.A.), or CPM‐V (Belotero Volume, 26 mg/mL HA; Anteis S.A.). All products in the CPM‐HA range contain 0.3% lidocaine except for CPM‐R.

The expert panel completed a pre‐meeting questionnaire regarding the medical management of these products and literature review on hybrid fillers. Two virtual discussions were then conducted and guided by a skilled moderator focusing on 10 topics: pre‐injection skin preparation, anesthetic use, hybrid fillers preparation (CPM‐HA choice, CaHA‐CMC:CPM‐HA ratio, technique, diluent, number of passes to mix the blend), syringe choice, injection protocol according to the anatomical zone (i.e., injection technique, injection plane, cannula size), duration of treatment, and possible complications/adverse events (side effects or complications that their patients have experienced when undergoing hybrid fillers injection, duration and treatment). Additionally, the expert panel discussed patient perspectives (i.e., “cost–benefit”, “whether clinical outcomes obtained with hybrid fillers justifies cost‐of‐treatments,” “whether hybrid use reduces treatment time (number of visits/interventions required)?”), treatment costs, demand for hybrid fillers in clinical practice, and future perspectives.

No formal consensus or Delphi panel was performed. Recommendations were described based on the percentage of the agreement among the respondents.

A descriptive analysis was performed to summarize expert thoughts on each topic. Percentages were determined based on the number of experts who responded to each item. Figures were created using GraphPad Prism software, version 6.01.

## 3 Results

Among the panel members, 50% (*n* = 5/10) have performed between 150 and 200 hybrid filler treatments, 20% (*n* = 2/10) between 100 and 150, 20% (*n* = 2/10) between 50 and 100, and 10% (*n* = 1/10) more than 200 hybrid filler treatments.

### 3.1 Recommendation 1: Preparation of the Skin Before the Hybrid Filler Application

Experts reported a similar process for skin preparation which involved an initial cleansing step (to remove any dirt, oil, or makeup) followed by the application of an antiseptic agent. Anesthesia –either topical or injectable—was applied next, followed by careful marking of the treatment areas (to guide the precise placement of the hybrid filler).

### 3.2 Recommendation 2: General Considerations for Filler Selection

The expert panel recommends that selection criteria for hybrid fillers should focus on the hybrid's rheological properties as well as the patient's specific treatment requirements. The patient factors considered highly relevant by most experts were: skin quality, soft tissue sagging, and the need for volume and/or projection. Age was also identified as a key variable, as many cutaneous aging processes tend to manifest at specific stages of life.

During clinical evaluation, experts emphasized the importance of adequate clinical assessment through skin palpation since sagging was one of the main features guiding their treatment decisions. In addition, clinical exam was required to identify elastosis and/or expression lines, hydration status, and facial morphology. Consultation with appropriate questioning was viewed as essential in identifying patients' expectations regarding treatment outcomes. Facial physiognomy must be carefully considered to maintain natural harmony and individualize treatment, particularly in populations with distinctive anatomical traits, such as the Mexican population.

### 3.3 Recommendation 3: Hybrid Filler Preparation

The expert panel formulated its recommendations based on scientific literature on hybrid fillers and their own clinical experience. The most common practice implemented by most of the panel members in their routine clinical settings involved several steps executed in a specific order; this helped to streamline the process and enhance functionality.

Firstly, a 10 mL syringe and a Luer‐lock connector are used to combine the contents of 1.5 1.5 mL of CaHA‐CMC with 0.5 mL of 2% lidocaine. This solution is then mixed with 1 mL of the chosen CPM‐HA. The mixture is then transferred via the Luer‐lock to an empty syringe of the same volume, and the contents are passed back and forth between the two syringes (20 times in total) to ensure thorough mixing. Some experts then apply the foaming technique to enhance the homogeneity of the mixture (1 to 2 passes).

Once the hybrid filler is fully mixed (homogeneous), the original CaHA‐CMC syringe is filled with the required quantity (depending on the specific anatomical zone being treated and the patient's needs).

#### 3.3.1 CaHA‐CMC Use

All the experts (100%, *n* = 10/10) preferred using the CaHA‐CMC without integrated lidocaine for the preparation of hybrid fillers but also reported using CaHA‐CMC with 0.3% powdered lidocaine hydrochloride (CaHA‐CMC (+); Radiesse (+); Merz North America Inc., Franksville, WI, USA) in certain instances (e.g., for mandibular contour enhancement).

Although most of the expert panel (70%, *n* = 7/10) prepare the hybrid filler in a single step by combining CaHA‐CMC, the diluent, and CPM‐HA simultaneously, 30% (*n* = 3/10) of experts preferred a two‐step process where CaHA‐CMC was first mixed with the diluent (which may include lidocaine and/or saline solution) and then combine this initial mixture with CPM‐HA.

##### 3.3.1.1 Recommendation 3.1: Use of Anesthetic and/or Saline Solution as Diluents in Hybrid Filler Preparation

Most of the experts (90%, *n* = 9/10) reported using 0.5 mL of 2% lidocaine; the remaining 10% (*n* = 1/10) reported using 0.3 mL. Although CPM‐HA already contains anesthetic, experts tend to add lidocaine as a diluent during the preparation of hybrid fillers, given that it acts as a fluidizing vehicle slightly reducing the viscosity of the mixture and improving its manageability, particularly during subcutaneous retroinjection and fanning techniques. There is no standardized final volume for the mixture, as each practitioner adjusts the degree of dilution based on the targeted clinical outcome.

Although formal rheological analyses are lacking, clinical experience suggests that higher proportions of lidocaine may reduce the viscosity of CaHA‐CMC, potentially influencing their injectability, spreadability, and tissue integration [10, 11].

Additionally, half of the experts reported using infiltrative anesthesia at the cannula entry points.

Sixty percent of the experts (*n* = 6/10) recommended adding saline solution to the mix of the hybrid filler. The reasons experts recommended this were “to make the product more diluted” (50%, *n* = 3/6), or, in the remainder of cases (50%, *n* = 3/6) “to have a greater final volume that facilitates distribution of the product across the entire face,” “to help the CaHA‐CMC microspheres spread,” and “to help the product mix better.” In contrast, 40% of the experts (*n* = 4/10), preferred not adding saline solution for the following reasons: “lidocaine is more practical to use,” “saline does not mix well in the final blend,” “the product should remain as concentrated as possible,” and “saline is not necessary since it works well with the HA mixture.”

##### 3.3.1.2 Recommendation 3.2: Number of Syringes Passes Required for Optimal Mixing

Fifty percent (*n* = 5/10) of the experts believed 10 to 15 passes between the syringes is sufficient to create a well‐mixed hybrid filler. The remaining 50% (*n* = 5/10) felt it is preferable to perform 20 to 30 passes to achieve a uniform and homogeneous final product. In the end, the panel unanimously agreed to calculate the average number of passes and standardize the number of syringe‐to‐syringe passes at 20.

With respect to the foaming technique, an even split (50%/50%) was initially observed regarding its use. However, after revisiting the available scientific evidence, 70% of the experts (*n* = 7/10) concluded that employing the technique is optimal considering that it facilitates better integration of the product into the tissue.

##### 3.3.1.3 Recommendation 3.3: Syringe Selection for Application of Hybrid Filler

All experts (100%, *n* = 10/10) confirmed using the CaHA‐CMC syringe when injecting the hybrid filler.

### 3.4 Recommendation 4: CPM‐HA Selection and Technique Based on Anatomical Region and Treatment Goals

Key points were explored for each anatomical region (face, neck, decolletage, and hands):

1. Choice of HA filler from the CPM‐HA portfolio.
  For instance, CPM‐R or CPM‐B are preferred for skin quality improvement (fine lines, hydration), often prepared with additional lidocaine or small amounts of saline to enhance distribution and minimize risk of nodules. For structural restoration and volume enhancement, combination with CPM‐V or CPM_I is recommended, using lower diluent volumes to maintain ability of projection.
2. Aesthetic objectives based on the patient's needs including reduction of fine lines, volume restoration, improved jawline definition, correction of sagging, and enhanced skin hydration.
3. The CaHA‐CMC:CPM‐HA ratio.
  For instance, the preparation of a 1:1 hybrid filler involves combining 1.5 mL of CaHA with 0.5 mL of lidocaine and 1 mL of a selected CPM‐HA, resulting in a total volume of 3 mL. This proportion ensures equal parts of CaHA and HA/diluent, thus achieving a true 1:1 ratio. Whenever a higher dilution is required, such as for thinner or atrophic skin, a 1:2 mixture can be prepared by combining 1.5 mL of CaHA with 2 mL of HA and 1 mL of lidocaine, yielding 4.5 mL in total. Conversely, for volumizing purposes, the amount of lidocaine is minimized (e.g., 0.1 mL) to reduce the burning sensations while preserving the product's rheological properties.
4. Injection technique, plane of injection and cannula size.

#### 3.4.1 Face

**FIGURE 1:**
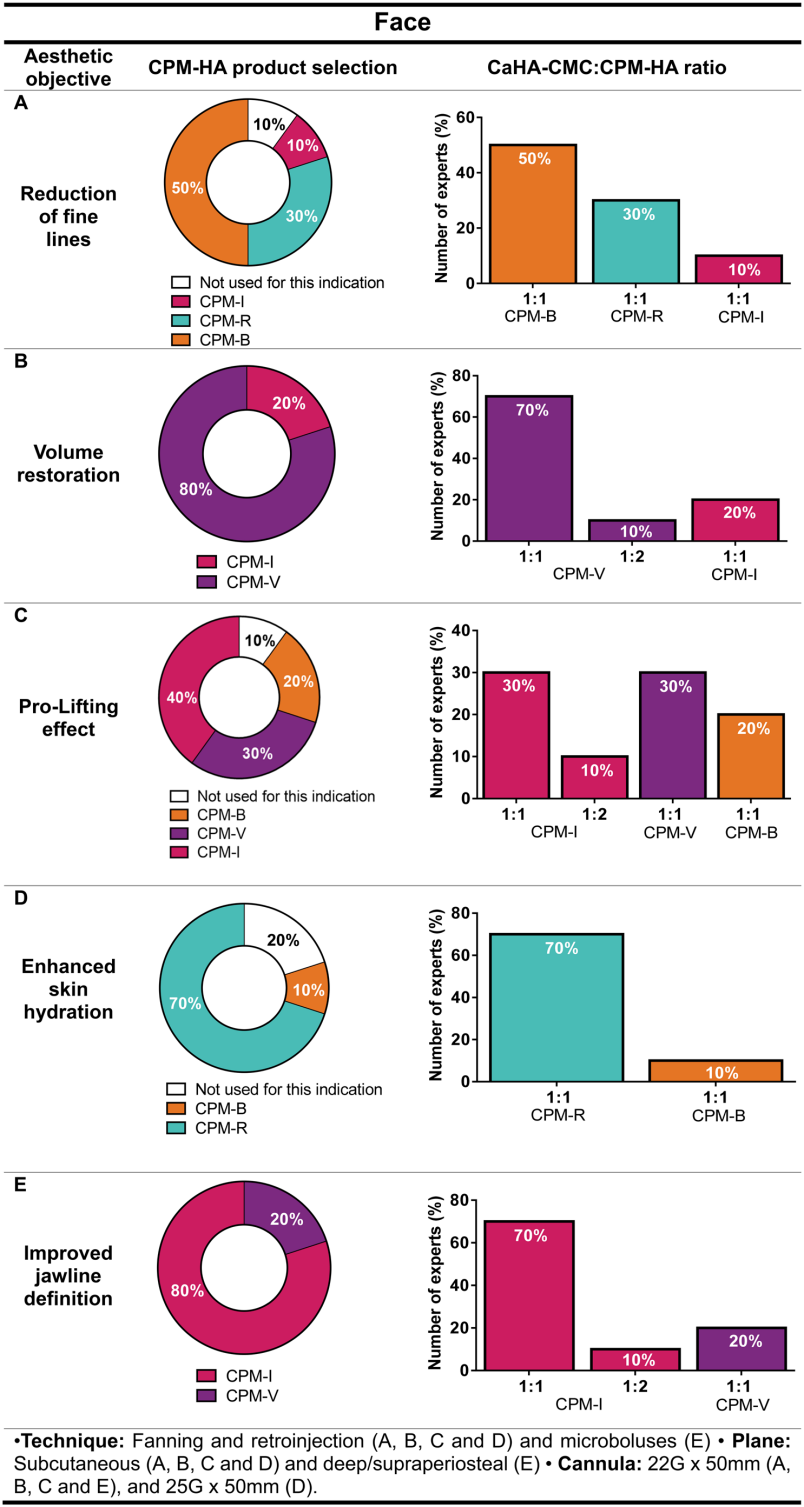
Recommendations on the selection of CPM‐HA products combined with CaHA‐CMC for facial aesthetic treatment, including suggested CaHA‐CMC:CPM‐HA ratios, injection technique, plane, and cannula size for (A) reduction of fine lines, (B) volume restoration, (C) pro‐lifting effect, (D) enhanced skin hydration, and (E) improved jawline definition. Pie charts indicate the percentage of experts selecting each CPM‐HA product; bar charts show the recommended CaHA‐CMC:CPM‐HA ratios. B, Balance; CaHA‐CMC, Calcium hydroxylapatite suspended in carboxymethylcellulose; CPM‐HA, Cohesive Polydensified Matrix hyaluronic acid; I, Intense; R, Revive; V, Volume.

Patients were considered candidates if they desired reduction of fine facial lines, especially if they had thin skin, visible lines, wrinkling, and mild sagging. As shown in Figure 1A, 50% of the experts (*n* = 5/10) reported using CPM‐B in combination with CaHA‐CMC, 30% (*n* = 3/10) CPM‐R, 10% (*n* = 1/10) CPM‐I and the other 10% (*n* = 1/10) do not use hybrid fillers for fine lines in the face. There was a unanimous agreement on the use of one syringe of CaHA‐CMC combined with one syringe of CPM‐HA (CPM‐B, CPM‐R and, CPM‐I; 1:1 dilution) for the treatment of facial fine lines.

Regarding volume restoration, 80% of the experts (*n* = 8/10) preferred using CPM‐V, while 20% (*n* = 2/10) favored CPM‐I as the HA filler. The most commonly used CaHA‐CMC:CPM‐HA ratio was 1:1, with only 10% of the experts (1/10) reporting the use of a 1:2 ratio when combining CaHA‐CMC with CPM‐V (Figure 1B).

Signs indicating the need for soft tissue pro‐lifting included loss of fat pads, lack of volume in the lateral zygomatic area, pronounced nasolabial folds (NLFs), and lack of definition/projection of the cheekbones. Regarding CPM‐HA selection, 40% (*n* = 4/10) of the experts selected CPM‐I, followed by CPM‐V (30%, *n* = 3/10), and CPM‐B (20%, *n* = 2/10). The remaining 10% (*n* = 1/10) reported not using hybrid filler for face pro‐lifting. Similarly to previous cases, most of the participants use a 1:1 ratio (Figure 1C).

According to the experts, a need for hydration improvement is usually required when the patient presents with loss of skin turgor (around the age of 30) or as a preventive measure to give the face a radiant glow. In this regard, 70% of the experts (*n* = 7/10) reported CPM‐R as their HA of choice, and 10% (*n* = 1/10) preferred CPM‐B. For both cases, the expert panel typically used them in a 1:1 ratio. On the other hand, 20% of the expert panel (*n* = 2/10) commented not to use hybrid fillers for this indication. (Figure 1D).

Additionally, in cases where the aesthetic objective was to improve jawline definition (Figure 1E), 80% of the expert panel (*n* = 8/10) reported preferring CPM‐I in a 1:1 or 1:2 ratio (70% and 10%, respectively), while 20% (*n* = 2/10) preferred using CPM‐V in a 1:1 ratio as the CPM‐HA gel for hybrids used for addressing this clinical need.

For most areas, fanning and retro‐injection techniques in the subcutaneous plane were preferred. Nonetheless, for mandibular profiling, 20% of the experts (*n* = 2/10) mentioned injecting microboluses in the deep/supraperiosteal plane as well. The 22G × 50 mm cannula was predominantly used among the experts for hybrid filler treatments targeting fine lines (50%, *n* = 5/10), volume restoration (50%, *n* = 5/10), pro‐lifting (50%, *n* = 5/10) and jawline definition (60%, *n* = 6/10) whereas for facial hydration (50%, *n* = 5/10), the preferred cannula size was 25G × 50 mm.

#### 3.4.2 Neck

**FIGURE 2:**
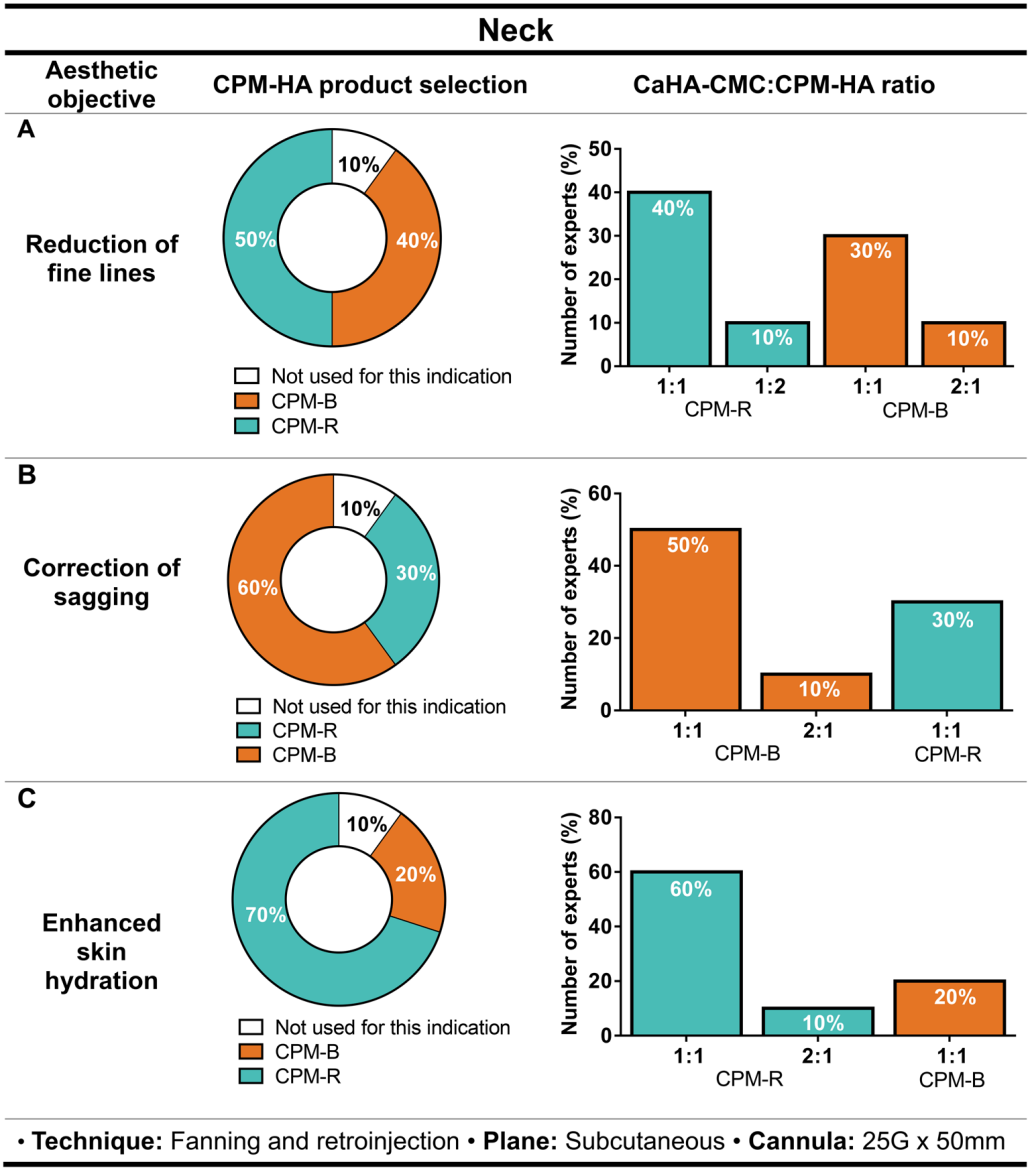
Recommendations on the selection of CPM‐HA products combined with CaHA‐CMC for neck aesthetic treatment, including suggested CaHA‐CMC:CPM‐HA ratios, injection technique, plane, and cannula size for (A) reduction of fine lines, (B) correction of sagging, and (C) enhanced skin hydration. Pie charts indicate the percentage of experts selecting each CPM‐HA product; bar charts show the recommended CaHA‐CMC:CPM‐HA ratios. CaHA‐CMC, Calcium hydroxylapatite suspended in carboxymethylcellulose; CPM‐HA, Cohesive Polydensified Matrix hyaluronic acid; R, Revive; B, Balance; I, Intense; V, Volume.

When addressing fine lines, 50% of the expert panel (*n* = 5/10) preferred the use of CPM‐R, 40% (*n* = 4/10) preferred CPM‐B, and 10% (*n* = 1/10) commented not to use hybrid fillers to address this need. The most recommended ratio was 1:1 for both CPM‐R (40%, *n* = 4/10) and CPM‐ B (30%, *n* = 3/10). Nonetheless, 1 expert (10%) mentioned using a 1:2 ratio for CPM‐R and a 2:1 ratio for CPM‐B (2 syringes of CaHA‐CMC and 1 syringe of CPM‐B) (Figure 2A).

For sagging correction, the selection of the appropriate CPM‐HA depended on the hydration of the skin and the evaluation of skin density. CPM‐B was the CPM‐HA most commonly used (60%, *n* = 6/10), followed by CPM‐R (30%, *n* = 3/10). Moreover, the CaHA‐CMC:CPM‐HA ratio of 1:1 was preferred when using CPM‐B (50%, *n* = 5/10) or CPM‐R (30%, *n* = 3/10). Only 10% (*n* = 1/10) reported using a 2:1 ratio (CaHA‐CMC:CPM‐B) (Figure 2B).

If the goal was to improve skin hydration in the neck, CPM‐R was the most widely adopted option, reported by 70% of the experts (*n* = 7/10), with a 1:1 CaHA‐CMC:CPM‐HA ratio (60%, *n* = 6/10; Figure 2C).

For these indications in the neck, all the experts confirmed that injection with fanning and retro‐injection techniques in the subcutaneous plane with a 25G × 50 mm cannula was preferred.

#### 3.4.3 Decolletage

In this anatomical zone, reaching an agreement among experts was challenging.

**FIGURE 3:**
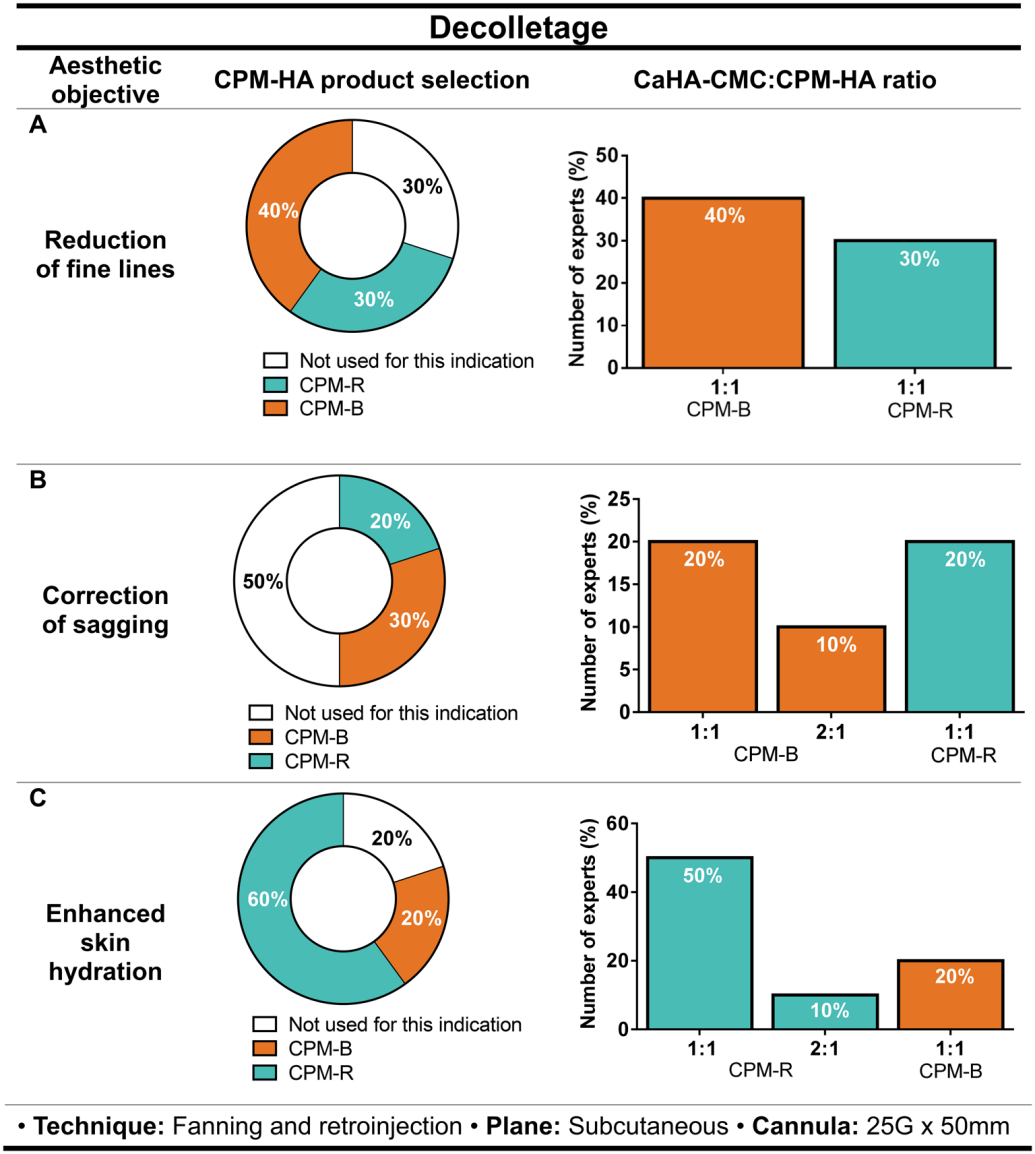
Recommendations on the selection of CPM‐HA products combined with CaHA‐CMC for decolletage aesthetic treatment, including suggested CaHA‐CMC:CPM‐HA ratios, injection technique, plane, and cannula size for (A) reduction of fine lines, (B) correction of sagging, and (C) enhanced skin hydration. Pie charts indicate the percentage of experts selecting each CPM‐HA product; bar charts show the recommended CaHA‐CMC:CPM‐HA ratios. B, Balance; CaHA‐CMC, Calcium hydroxylapatite suspended in carboxymethylcellulose; CPM‐HA, Cohesive Polydensified Matrix hyaluronic acid; I, Intense; R, Revive; V, Volume.

In the case of reducing fine lines, 40% of the experts (*n* = 4/10) use CPM‐B, 30% (*n* = 3/10) use CPM‐R, while the remaining 30% (*n* = 3/10) do not use hybrid fillers for this indication given that they prefer to use CaHA‐CMC alone (Figure 3A).

The use of hybrid fillers for sagging correction on the decolletage was not the first choice for half of the experts (50%, *n* = 5/10), while for the remaining 30% (*n* = 3/10) and 20% (*n* = 2/10), the HA fillers of choice for the hybrids blend were CPM‐B and CPM‐R, respectively (Figure 3B).

When considering the use of a hybrid filler for decolletage hydration, experts evaluated the presence of dynamic wrinkles and dehydrated skin. Most experts (60%, *n* = 6/10) preferred mixing CaHA‐CMC with CPM‐R, whereas 20% (*n* = 2/10) mentioned using CPM‐B for the hybrid blend. The remaining 20% (*n* = 2/10) reported not using hybrid fillers for this indication, preferring the use of CPM‐HA alone (Figure 3C). Experts also mentioned that for a preventive treatment only, using a combination of CaHA‐CMC and CPM‐R would be suitable to improve skin density.

Similarly to the neck, the 1:1 CaHA‐CMC:CPM‐HA ratio, injection with a 25G × 50 mm cannula, using fanning and retro‐injection techniques at the subcutaneous level was recommended.

#### 3.4.4 Hands

**FIGURE 4:**
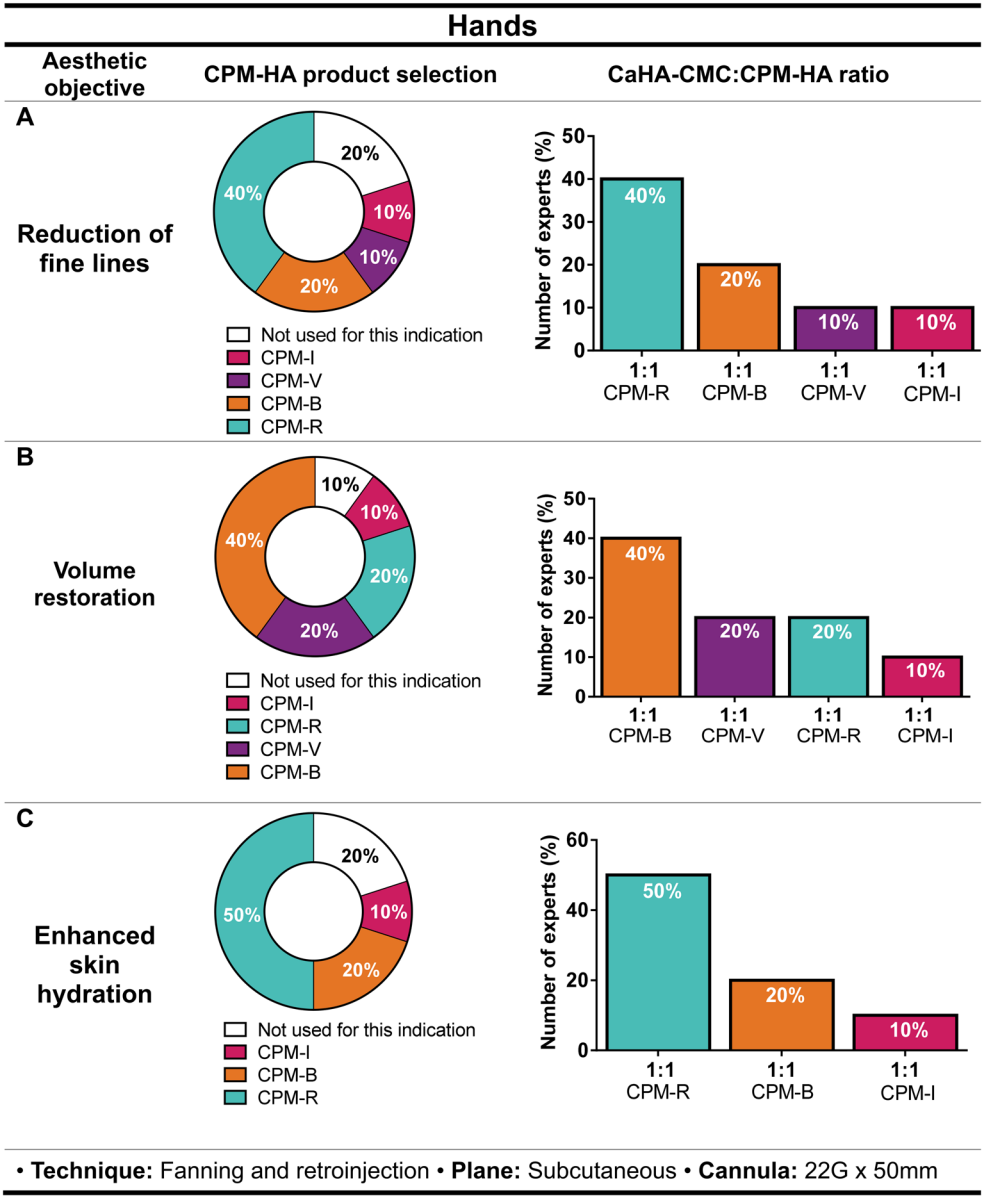
Recommendations on the selection of CPM‐HA products combined with CaHA‐CMC for hand aesthetic treatment, including suggested CaHA‐CMC:CPM‐HA ratios, injection technique, plane, and cannula size for (A) reduction of fine lines, (B) volume restoration, and (C) enhanced skin hydration. Pie charts indicate the percentage of experts selecting each CPM‐HA product; bar charts show the recommended CaHA‐CMC:CPM‐HA ratios. Pie charts indicate the percentage of experts selecting each CPM‐HA product; bar charts show the recommended CaHA‐CMC:CPM‐HA ratios. B, Balance; CaHA‐CMC, Calcium hydroxylapatite suspended in carboxymethylcellulose; CPM‐HA, Cohesive Polydensified Matrix hyaluronic acid; I, Intense; R, Revive; V, Volume.

For treating fine lines, experts believe it is important to consider skin thickness (easily assessed by asking the patient to move the hand to better observe dynamic wrinkles). Preferred CPM‐HA for the hands varied between CPM‐R (40%, *n* = 4/10), CPM‐B (20%, *n* = 2/10), CPM‐V (10%, *n* = 1/10) and CPM‐I (10%, *n* = 1/10), while 20% (*n* = 2/10) confirmed not to use hybrid fillers for this purpose (Figure 4A).

The best indication that volume is needed would be when tendinous lines, blood vessels, visible bones, or the presence of devitalized skin. The choice of product from the CPM‐HA range was CPM‐B (40%, *n* = 4/10), CPM‐V (20%, *n* = 2/10), CPM‐R (20%, *n* = 2/10), and CPM‐I (10%, *n* = 1/10). The remaining 10% (*n* = 1/10) commented not to use hybrid fillers for this indication. (Figure 4B).

Key indicators of the need for hand hydration included persistent skin dryness despite the use of moisturizing creams and age over 40 years. In this case, CPM‐R (50%, *n* = 5/10) was the preferred choice of CPM‐HA for the blend, followed by CPM‐B (20%, *n* = 2/10) and CPM‐I (10%, *n* = 1/10), whereas 20% of the experts (*n* = 2/10) do not use hybrid fillers for this indication.

Regardless of the aesthetic objective, there was a consensus on the use of a CaHA‐CMC:CPM‐HA 1:1 ratio, fanning and retro‐injection techniques and injection into the dorsal superficial lamina for treating fine lines and improving hydration, and into the dorsal intermediate lamina for addressing volume loss, using a 22G × 50 mm cannula (Figure 4C).

### 3.5 Duration of Results of Hybrid Fillers

According to the clinical experience of the expert panel, 60% (*n* = 6/10) confirmed that once the hybrid filler is applied, the duration of the treatment lasts for more than 12 months, 20% (*n* = 2/10) reported a 12‐month duration and 20% (*n* = 2/10) from 6 to less than 12 months.

### 3.6 Adverse Events (AEs) of Hybrid Fillers

To explore the safety profile of hybrid fillers based on clinical practice in Mexico, the expert panel was asked to report the adverse events (AEs) observed in their patients. The most frequently mentioned AEs were hematoma and edema (80%, *n* = 8/10), followed by nodules due to product accumulation (50%, *n* = 5/10), inflammation and pain (40%, *n* = 4/10), and finally erythema and sensitivity (20%, *n* = 2/10).

However, despite being frequently cited by the experts, these AEs were reported to occur at low prevalence among treated patients. According to expert estimation, inflammation was the most prevalent AE (50%), followed by edema (40%) and erythema (20%). Nodules, pain, and sensitivity were each reported in only 14% to 10% of patients. Duration varied depending on the AE, with most resolving within 3 to 5 days, except for hematoma (5–7 days) and nodules, which showed treatment‐dependent resolution (Table 1).

**TABLE 1:** Adverse events, estimated prevalence, duration, and recommended management following hybrid treatment with CaHA‐CMC combined with CPM‐HA based on expert panel insights.

AE	Estimated prevalence of AE in patients treated with CaHA‐CMC + CPM‐HA (%)	Duration (days)	Recommended management
Inflammation	50	3–5	No treatment required
Edema	40	3–5	No treatment required
Erythema	20	5	No treatment required
Hematoma	17	5–7	Option 1: Serratio peptidase (10 mg), 2 tablets every 8 h for 5 days Option 2: Arnica
Nodules (product accumulation)	14	Variable; treatment‐dependent resolution	Option 1: Saline solution with 2% lidocaine ± ultrasound Option 2: Collagenase with lidocaine 2% and massage
Pain	10	3–5	No treatment required
Sensitivity	10	1–3	No treatment required

Abbreviation: AE, Adverse event.

Inflammation (50%), edema (40%), and erythema (20%) were the most commonly referred AEs, typically resolving within 3 to 5 days without the need for intervention. Hematomas were estimated to occur in 17% of treated patients; recommended management for this AE includes serratio peptidase or arnica.

Nodules related to product accumulation (estimated in 14% of patients) were managed with either saline solution and 2% lidocaine (± ultrasound) or collagenase with massage. Pain and sensitivity were less frequent (10% each) and resolved spontaneously. Most AEs were mild and self‐limited, requiring minimal or no treatment.

### 3.7 Cost–Benefit Ratio of Hybrid Fillers

Experts believed that the cost was justified to patients given the clinical outcomes obtained either “totally” (50%, *n* = 5/10 of experts) “mostly” (40%, *n* = 4/10) or “moderately” (10%, *n* = 1/10). Ninety percent of the panel (*n* = 9/10) agreed that the use of hybrids enables the reduction of treatment time (number of visits/interventions required).

### 3.8 Demand and Trend of Hybrid Fillers

The main factors that have driven the adoption of hybrid treatments in clinical practice were identified based on the frequency that each factor was ranked as the most important by the experts. High patient satisfaction was selected as the top factor by 50% of respondents (*n* = 5/10), followed by clinical efficiency (30%, *n* = 3/10), technological innovation (10%, *n* = 1/10), and prolonged duration of results (10%, *n* = 1/10).

All experts unanimously agreed that patients are not familiar with hybrid fillers, making it essential for the clinician to clearly explain the indication, benefits, and potential risks. The panel reached full consensus on the pivotal role of medical recommendation, identifying it as the most influential factor in a patient's decision to undergo hybrid filler treatment.

Patients' main expectations included long‐lasting rejuvenation, natural‐looking results, and minimal post‐treatment downtime.

## 4 Discussion

While the popularity of hybrid fillers in aesthetic medicine is increasing, and standardized protocols for their use in aesthetic medicine have been previously published [4], anatomical and clinical differences among populations may limit their direct applicability. The present recommendations were conducted to address these considerations and to provide tailored guidance for the clinical application of hybrid fillers in the Mexican population. Combining the expertise of key opinion leaders in Mexico and available scientific evidence, a series of recommendations were established for specific areas, such as the face, neck, décolletage, and hands for their use in the Mexican population.

Mexico ranks among the top three Latin American countries performing the highest number of surgical and non‐surgical aesthetic procedures [12]. Across Latin America (LA), there is significant ethnic diversity, with Mestizos (of mixed Spanish and Indigenous ancestry) representing the majority population in approximately half of the countries in the region [13]. In addition to Mestizos, Mexico exhibits a high degree of Native American ancestry in the central and southern regions, whereas northern populations tend to have a greater proportion of European ancestry [14].

The mix of diverse ethnic backgrounds in LA contributes to a wide range of skin tones, facial morphologies, bone structures, tissue characteristics, and fat distribution. These differences not only impact anatomical considerations but also influence patient preference and the onset of visible aging. For instance, Hispanic populations favor fuller lips contrasting with Eastern Asian populations [15]. Mexican women have described beauty as smooth skin, clean, healthy, natural and unique skin [16]. Moreover, aging signs tend to appear later in Hispanics than in Caucasians, typically after their fifth decade of life [17]. Structural traits like a thicker dermis may contribute to this delayed wrinkle formation. Nonetheless, Hispanic skin, particularly among individuals with darker phototypes, is more susceptible to post‐inflammatory hyperpigmentation due to increased melanocyte activity and melanin content [18, 19].

The average Mexican patient may exhibit facial features such as prominent malar eminences, a wider bizygomatic distance resulting in a broader and heavier facial appearance, midface flattening due to reduced maxillary projection, increased submental fat or fullness, a broad nose with a widened alar base, a short columella, horizontally oriented nostrils, and thick nasal skin [20, 21]. Common aesthetic concerns reported by Latin American populations include infraorbital hollowness and darkening, deep nasolabial folds, jowl formation, and inadequate chin projection [22].

The panel confirmed that hybrid fillers align with the main expectations patients have for minimally invasive aesthetic treatments, including long‐lasting rejuvenation, natural‐looking results, and minimal post‐treatment recovery. Nonetheless hybrid fillers offer additional advantages: they can reduce treatment time, enhance the patient experience by requiring fewer injections, and address a broader range of aesthetic concerns in a single session, as the combined fillers may offer complementary mechanisms of action. For example, with the CaHA‐CMC:CPM‐HA hybrid, the CPM‐HA component renders an immediate volumizing effect, compensating for the early volume loss secondary to the resorption of the carboxymethylcellulose gel carrier of CaHA‐CMC, when the CaHA‐CMC‐induced neocollagenesis has not yet taken place [1]. On the other hand, the ECM regenerating properties of CaHA‐CMC not only extend the duration of the filler treatment in the long‐term [2] avoiding the loss of volume that usually occurs as CPM‐HA is degraded, but also enhance the quality of the skin by providing “skin glow.” Moreover, the combination of CaHA‐CMC and CPM‐HA improves the administration of the fillers, since the resulting gel is more pliable and moldable compared to CaHA‐CMC alone [4]. Since CaHA‐CMC exhibits limited hygroscopic power compared to CPM‐HA, the risk of frequent edema, compared to CPM‐HA injected alone, may be lowered [23].

A common aspect observed in Mexican patients, the so‐called “heavy” face related to wide bizygomatic distance, thicker skin, and greater soft tissue volume, is associated with a tendency toward gravitational descent. In the latter scenario, the association of high G' (elastic modulus) HA (i.e., CPM‐I or CPM‐V) and CaHA‐CMC in the hybrid filler enhances the tissue elevation capacity in dynamic compression of CPM‐HA [5], especially in areas with non‐distensible tissue or high soft‐tissue volume and weight where the lifting potential of the filler must be great enough to provide sufficient projection (e.g., malar, temple) [24, 25]. CaHA‐CMC ranks among the highest viscosity, elastic modulus in shear (G') and normal stress (E') among fillers, thus ensuring that it efficiently withstands gravitational forces, providing composite lift, and support to the overlying tissues [24].

The panel highlighted that the hybrid treatment approach should follow the same rules for other fillers: there is no one‐size‐fits‐all, and the treatment should be tailored to the patient's needs and expectations. Several types of CPM‐HA can be utilized in this procedure, offering versatility, treatment effectiveness, and patient safety [26]. Each CPM‐HA filler has been manufactured with specific crosslinking parameters and HA concentrations, resulting in different rheological properties [27] appropriate for use in distinct layers (superficial or deep) and for different purposes (e.g., lifting, volumization, definition) [28]. For instance, CPM‐HA fillers with lower viscosities can be administered superficially with homogeneous integration and optimal spreading, similar to naturally occurring HA [29], whereas CPM‐HAs with high viscosities and high E' provide an optimal capacity for tissue projection and resistance to dynamic forces in deeper layers [30, 31]. As per expert panel, the choice of hybrid filler should be guided by skin quality (e.g., degree of elastosis, hydration status), degree of sagging, volume loss, underlying anatomy, and the patient's expectations. For instance, in many regions of Mexico, patients commonly experience dry skin [16], and intense sun exposure. These individuals may particularly benefit from the inclusion of CPM‐R in the hybrid filler, due to its hydrophilic properties. On the other hand, when addressing common features of the Mexican patients, such as rounder faces and retracted chin, the association of CaHA‐CMC with high viscosity and E' HAs (e.g., CPM‐I or CPM‐V) is required for soft‐tissue support (e.g., chin projection) or definition (e.g., mandibular profiling). In a 5‐year retrospective analysis of hybrid filler treatment, CPM‐I was solely used for mandibular profiling, while in the current study, besides mandibular profiling, experts also referred to its use for facial soft tissue lifting, loss of facial volume, combined wrinkling and mild sagging in the face and in the hands [3].

Although no formal consensus methods (e.g., Delphi panel) were performed, interestingly the fanning and retro‐injection techniques, and use of cannula 25G x 50 mm (for face, neck and décolletage) and 22G × 50 mm (for hands) were overwhelmingly preferred across the different anatomical regions. CaHA‐CMC:CPM‐HA dilution ratio preference in the current study was 1:1, regardless of the type of CPM‐HA, corroborating previous studies. In a retrospective cohort of 2112 patients, the average ratio was reported as 0.63, with 2:1 (CaHA‐CMC:CPM‐HA) as the highest and 1:5 as the lowest, with the most used ratio as 1:1 for all CPM‐HA types except CPM‐HA B (1:2) [3].

For some topics, reaching a consensus was harder. For instance, the use of saline solution was controversial among the experts, with some advocating for its inclusion to improve distribution and homogeneity, while others preferred maintaining a higher product concentration for better efficacy. Additionally, while most experts preferred a single‐step dilution method, a notable proportion opted for a two‐step dilution, suggesting that individual preferences and clinical experience also influence preparation techniques.

Hybrid filler complications were recently classified as *early/injection‐related* (erythema, edema, vascular events) versus *late* (nodules, granulomas), stressing that nodules remain the most relevant long‐term complication [32]. The panel highlighted some preventive and management strategies for adverse events with hybrid fillers, particularly focusing on vascular safety: use of 22G blunt cannulas, slow injection avoiding pressure, pre‐treatment ultrasound imaging to avoid intravascular injection, and maintenance of an emergency kit with hyaluronidase [33]. Experts also noted that early nodules are most linked to insufficient dilution, whereas late nodules may potentially be biofilm‐related. Additionally, nodules are more likely to occur in thin or atrophic skin (e.g., neck, dorsal hands), and that hyperdilution [34] or selection of lower‐viscosity CPM‐HA (e.g., CPM‐R or CPM‐B) may reduce this risk. Treatment with massage, ultrasound, or hyaluronidase would depend on the baseline etiology. These clinical insights are consistent with published data from a multicenter retrospective analysis of a hybrid HA/CaHA filler that adverse events were rare (< 3%), most being mild and transient, with occasional cases of edema, induration, or nodularity, all resolving without sequelae [35].

At present, clinical experience and patient outcomes serve as the primary sources of knowledge. In routine clinical practice, outcome assessment has been primarily based on the comparison of pre‐ and post‐treatment clinical photographs, complemented by the patient's subjective perception of satisfaction (commonly categorized as good, fair, or poor). However, use of validated scales, particularly the Merz Aesthetics Scale for skin laxity, which can be employed as a reference tool in clinical practice to objectively assess aesthetic improvement.

Further research is needed to address questions from the panel, such as changes in the rheology of the fillers within the blend, impact on the individual products in combination, impact on glycerol, rheology of different dilution ratios, comparison of the longevity of hybrid fillers prepared with different dilutions, as assessed by magnetic resonance imaging, or computed tomography. This limited data may hamper the ability to precisely predict in vivo behavior and to standardize application techniques. Available data are limited on describing how their physicochemical properties are modified when blended [36]. Key rheological characteristics such as viscosity, cohesivity, G', and E' remain yet to be objectively evaluated. According to the expert panel, dilution strategies are critical in hybrid fillers, especially when combining CaHA‐CMC with CPM‐HA. Since CaHA‐CMC can be rheomodulated through graded dilution, shifting its viscoelastic properties across a wide spectrum comparable to HA fillers [37], the panel supports tailoring the dilution by anatomical site and skin thickness. For thinner or atrophic skin (e.g., neck or dorsal hands) increasing dilution (e.g., adding 0.5 mL of saline or selecting lower‐viscosity CPM‐HA products such as CPM‐R or CPM‐B) minimizes the risk of nodularity and renders a more homogeneous tissue distribution. Conversely, for thicker skin requiring volumization, CPM‐ I or CPM‐V may be selected, with reduced dilution to preserve lifting capacity.

Moreover, generating data in diverse populations is especially relevant for the Mexican population, given the specific anatomical features, skin types, and aging patterns that distinguish it from other populations.

## 5 Conclusion

As the use of hybrid fillers continues to grow within clinical practice, and its safety and efficacy have been shown for different areas and indications, this document underscores the importance of tailoring the treatment approach to achieve optimal results and meet patients' expectations, especially considering diverse populations such as the Mexican, whose anatomical, genetic, and ethnic characteristics differ significantly from those typically represented in global studies.

## Author Contributions

All authors made a significant contribution to the work reported, whether that is in the conception, study design, execution, acquisition of data, analysis and interpretation, or in all these areas; took part in drafting, revising or critically reviewing the article; gave final approval of the version to be published; have agreed on the journal to which the article has been submitted; and agree to be accountable for all aspects of the work.

## Ethics Statement

The authors confirm that the ethical policies of the journal, as noted on the journal's author guidelines page, have been adhered to. No ethical approval was required as this is a review article with no patient data.

## Conflicts of Interest

The authors Garcia and Zavaleta are LATAM consultants for Merz Aesthetics. Arenas, Ávila, Baca, Torres García, Balbas, Vilchez, Vázquez, Lozano are consultants for Merz Aesthetics Mexico (Merz Pharma S.A. de C.V.), and Fakih‐Gomez is a consultant for Global Merz Aesthetics.

## Data Availability

The data that support the findings of this study are available from the corresponding author upon reasonable request.
